# MASH1/Ascl1a Leads to GAP43 Expression and Axon Regeneration in the Adult CNS

**DOI:** 10.1371/journal.pone.0118918

**Published:** 2015-03-09

**Authors:** Ryan R. Williams, Ishwariya Venkatesh, Damien D. Pearse, Ava J. Udvadia, Mary Bartlett Bunge

**Affiliations:** 1 The Miami Project to Cure Paralysis, University of Miami Miller School of Medicine, Miami, FL, United States of America; 2 Department of Biological Sciences, University of Wisconsin-Milwaukee, Milwaukee, WI, United States of America; 3 Department of Neurological Surgery, University of Miami Miller School of Medicine, Miami, FL, United States of America; 4 Department of Cell Biology, University of Miami Miller School of Medicine, Miami, FL, United States of America; Biogen Idec, UNITED STATES

## Abstract

Unlike CNS neurons in adult mammals, neurons in fish and embryonic mammals can regenerate their axons after injury. These divergent regenerative responses are in part mediated by the growth-associated expression of select transcription factors. The basic helix-loop-helix (bHLH) transcription factor, MASH1/Ascl1a, is transiently expressed during the development of many neuronal subtypes and regulates the expression of genes that mediate cell fate determination and differentiation. In the adult zebrafish (Danio rerio), Ascl1a is also transiently expressed in retinal ganglion cells (RGCs) that regenerate axons after optic nerve crush. Utilizing transgenic zebrafish with a 3.6 kb GAP43 promoter that drives expression of an enhanced green fluorescent protein (EGFP), we observed that knock-down of Ascl1a expression reduces both regenerative gap43 gene expression and axonal growth after injury compared to controls. In mammals, the development of noradrenergic brainstem neurons requires MASH1 expression. In contrast to zebrafish RGCs, however, MASH1 is not expressed in the mammalian brainstem after spinal cord injury (SCI). Therefore, we utilized adeno-associated viral (AAV) vectors to overexpress MASH1 in four month old rat (Rattus norvegicus) brainstem neurons in an attempt to promote axon regeneration after SCI. We discovered that after complete transection of the thoracic spinal cord and implantation of a Schwann cell bridge, animals that express MASH1 exhibit increased noradrenergic axon regeneration and improvement in hindlimb joint movements compared to controls. Together these data demonstrate that MASH1/Ascl1a is a fundamental regulator of axonal growth across vertebrates and can induce modifications to the intrinsic state of neurons to promote functional regeneration in response to CNS injury.

## Introduction

Upon CNS injury, adult teleosts display a robust regenerative response that leads to functional recovery [1]. Although adult mammalian neurons have limited regenerative ability, when provided with a supportive environment they can regenerate axons across sites of CNS injury [2]. Modifications to the intrinsic growth state of injured neurons can further enhance regenerative ability [3–5]. Unlike adult neurons, developing mammalian neurons regenerate axons after injury [6] and also grow axons when transplanted into the injured adult CNS [7]. However, during development, neurons undergo a transcriptionally regulated temporal switch that limits their regenerative capacity [8–14]. Whereas both mammals and teleosts undergo a developmental switch in which growth-associated genes are transcriptionally silenced, in fish many of these genes are reactivated upon CNS injury.

Reactivation of growth-associated genes during axon regeneration in zebrafish requires enhancer elements that are distinct from those used during development [15–17]. The conserved, regeneration-specific enhancer regions in fish homologues of the growth-associated protein 43 (GAP43) [18] include a putative binding site for the evolutionarily conserved pro-neuronal transcription factor, MASH1. In mammals, MASH1 is transiently expressed in the developing retina [19] and brain [20] and directly regulates genes involved in axon growth [21]. Furthermore, MASH1/Ascl1a is re-expressed after zebrafish CNS injury [22–23] but not mammalian CNS injury.

We investigated the importance of Ascl1a in zebrafish CNS axon regeneration using morpholinos to knockdown Ascl1a expression in RGCs after optic nerve transection in transgenic zebrafish *gap43* reporter lines. Unlike mammalian RGCs, zebrafish RGCs survive optic nerve transection and regenerate injured axons [24–25]. We demonstrate that knockdown of Ascl1a expression in RGCs after optic nerve transection results in a reduction of both *gap43* gene expression and axon regeneration.

To test the efficacy of MASH1 expression in mammalian CNS regeneration we used adeno associated virus (AAV) vectors to ectopically express MASH1 in brainstems of four month old, adult rats, which do not normally re-express MASH1 in response to injury. After mammalian SCI, the transplantation of Schwann cells (SCs) provides a permissive environment for axon regeneration and is currently being evaluated clinically (www.clinicaltrials.gov NCT01739023). Here we demonstrate that following complete spinal cord transection and SC bridge implantation, rats whose brainstems were transduced with AAV-MASH1 exhibited both increased noradrenergic axon regeneration into the bridge and improvement in hind limb locomotion. Together, these experiments suggest that MASH1 expression is an evolutionarily conserved mechanism necessary for axon regeneration in teleosts that can be ectopically induced in mammals to promote regeneration and offers a potential therapeutic target for the treatment of patients with CNS injury.

## Methods

### Zebrafish husbandry and reporter lines

Zebrafish husbandry and all experimental procedures were approved by the Institutional Animal Care and Use Committee (IACUC) at the University of Wisconsin-Milwaukee. Zebrafish colonies were maintained as previously described [18]. Adult zebrafish were maintained at 28°C with 14/10 light/dark cycle and fed twice daily with Artemia as well as Zeigler Adult Zebrafish Complete Diet (VWR, West Chester, PA). Two strains of zebrafish were used in these experiments. A transgenic reporter strain constructed on the Ekkwill background, Tg (*Tru*.*gap43*:*egfp*) mil1, a.k.a. *fgap43*:*egfp* [26] was used for the *gap43* gene expression studies (n = 8), and a wild type strain (Ekkwill) was used for the RGC axon regeneration assays (n = 18).

### Zebrafish optic nerve injury and morpholino (MO) treatments

All zebrafish experiments were approved by Institutional Animal Care and Use Committee (IACUC) at the University of Wisconsin-Milwaukee and were performed in accordance with animal welfare standards established by the USA National Institutes of Health guide for the care and use of laboratory animals. For the gene expression assays, 6–9 month old *fgap43*:*egfp* zebrafish were anesthetized with 0.03% aminobenzoic acid ethylmethylester (Argent Chemical Labs, Redmond, WA), and their left optic nerves were fully transected one mm from the retina. The right eyes were left intact to serve as unoperated controls. Gene knockdown was accomplished by placing a small piece of gel foam soaked with MOs (Gene tools LLC, Philomath, OR) at the site of optic nerve transection. Three types of MOs were used: 1) a Ascl1a MO as previously described (n = 4) [27], 2) a negative control MO that does not target any zebrafish genes (n = 4) [28], and 3) The gap43 transgene used in the study contains the 5’ end of *gap43* along with exon 1 that encodes the first 10 amino acids of the protein. The resulting transgene encodes an EGFP fusion protein that is targeted by the GAP43 morpholino (S1 Fig.). The sequence used for morpholino synthesis is: GAP43 (start site) 5’ TCTTCTGATGCAGCACAGCATAGTC 3’. All of the MOs were tagged with the red fluorescent tracer, lissamine. This allowed for identification of neurons that received MOs through retrograde transport. Four days post injury animals were sacrificed, and retinas were removed, fixed, and prepared for frozen sectioning as previously described [18]. Transverse sections (10 μm) were collected on glass slides and cover slipped in Vectashield mounting medium (Vector Labs, Burlingame, CA) with DAPI to stain the nuclei. Fluorescent images were obtained using a Zeiss Apotome microscope. Cells expressing *gap43* were identified by the green fluorescence from the fugu *gap43*:*egfp* transgene [26].

For the RGC axon regeneration assays, 6–9 month old wild type zebrafish were anesthetized and their left optic nerves were partially transected one mm from the retina. Then a piece of gel foam soaked with Ascl1a (n = 6), GAP43 (n = 6), or negative control (n = 6) MOs was placed at the site of transection. Most of the gelfoam was dissolved by four days. After four days, remaining gelfoam was removed and a complete transection was performed one mm distal to the original injury, into which gel foam soaked with green fluorescent retrograde tracer (Alexa 488 conjugated dextran, Invitrogen, Grand Island, NY) was positioned to identify RGCs that had regenerated their axons. Three days after retrograde tracing, retinas were removed and prepared for frozen sectioning and fluorescence microscopy as described above. Images were collected from five sections per retina, which included sections from the center of the retina containing the optic nerve and two lateral sections on each side. RGCs that were injured and received the MO from the first transection were identified by the red fluorescence from lissamine. RGCs that regenerated their axons one mm past the original injury site were identified by green fluorescence from the tracer. The percentage of axon regeneration was calculated by counting the number of double fluorescent cells (yellow), divided by the total number of cells taking up the MO and multiplied by 100.

### Quantitative RT-PCR

To assess for relative changes in gene expression, quantitative PCR (qPCR) was performed on an ABI 7500 Fast Real time PCR system (Applied Biosystems, Carlsbad, CA) with SYBR green fluorescent label (Quanta Biosciences, Gaithersburg, MD). Total RNA was isolated from adult retina of *fgap43*:*egfp* zebrafish using Trizol reagent (Invitrogen, Grand Island, NY). The cDNA was synthesized from 550 ng total RNA with Oligo dT priming using qSCRIPT reverse transcriptase (Quanta Biosciences, Gaithersburg, MD). The qPCR analysis was performed to determine the relative levels of *gap43* and *gfp* mRNA in each sample, using *ef1α* as an internal control. Primer sequences used for each gene are as follows: *gap43* 5’- CCAAAGAGGAAGTGAAGGAG-3’ and 5’-CAGCAGCGTCTGGTTTGTC-3’; *gfp* 5’-AACGAGAAGCGCGATCAC-3’ and 5’-CCATAGGTTGGAATCTTAGAG-3’; *ef1α* 5’- GTACTTCTCAGGCTGACTGTG-3’ and 5’-CGCTGACTTCTTGGTGAT-3’. A dissociation step was performed at the end of the amplification phase to confirm a single, specific melting temperature for each primer set. Cycle threshold values (Ct) were normalized to *ef1α* as an internal reference. Relative gene expression was quantified using the 2^-(ΔΔCt) method [29], ΔCt1 = Normalized Ct (operated Lt eye) and ΔCt2 = Normalized Ct (unoperated Rt eye). A similar analysis was performed on retinas treated with negative control MOs for comparison. Normalized gene expression data from 3–4 biological replicates were averaged and analyzed as fold change.

### Rat husbandry and experimental design

Rat husbandry and all experimental procedures were approved by the Institutional Animal Care and Use Committee at the University of Miami and were performed in accordance with animal welfare standards established by the USA National Institutes of Health guide for the care and use of laboratory animals. The experimental design and methods used in this study have been previously described in detail by Williams and colleagues [30]. Briefly, twenty-one young adult female Fischer rats, obtained from Harlan Laboratories (Frederick, MD), each received two separate surgeries: first, the stereotaxic injection of AAV vectors and second, the complete transection of the spinal cord with the implantation of a SC bridge. In order to allow AAV vectors to form concatamers and achieve maximal transgene expression at the time of injury, the stereotaxic surgery was performed between two and six weeks prior to injury. The rats were twelve weeks of age and weighed 160–180 g at the time of injury. Their hindlimb locomotion was assessed weekly, and six weeks after injury they were perfused for histological analysis. All surgical procedures and analyses were performed blinded to the experimental group.

### AAV vectors

Serotype 2 AAV vectors were generated by the Miami Project to Cure Paralysis Viral Vector Core, using the AAV Helper-Free System from Stratagene (La Jolla, CA). The transgene plasmids for enhanced EGFP and MASH1 were kindly provided by Dr. Scott Whittemore (University of Louisville, KY) and Dr. David Anderson (California Institute of Technology), respectively. The AAV titers were determined to be 2.6 x 10^8^ TU/mL and 1.0 x 10^8^ TU/mL for AAV-EGFP and AAV-MASH1, respectively. The control group (n = 11) received AAV-EGFP and the treatment group (n = 10) received a 1:1 mixture of AAV-EGFP and AAV-MASH1.

### Stereotaxic injection of AAV

Detailed stereotaxic coordinates and injection procedures were described previously by Williams and colleagues [30]. Briefly, rats were anesthetized by intraperitoneal injection of ketamine (45 mg/kg; Vedco, St. Joeseph, MO) and xylazine (5 mg/kg; Acorn, Decatur, IL). Then stereotaxic injections were performed bilaterally, to target descending noradrenergic brainstem populations in the locus coeruleus, ventro-lateral pons, and the ventro-medial rostral medulla. A total of 19 μL of AAV was injected into each animal using a sp310i syringe pump (World Precision Instruments, Sarasota, FL) together with a 5 μL syringe modified with a 5 cm 33GA removable needle (Hamilton Company, Reno, NV) at a rate of 0.25 μL per minute.

### Generation of purified SCs

Purified (95–98%) SC populations were obtained from adult female Fischer rat sciatic nerves as described previously [31–32]. Following purification, SCs were passaged to confluency three times and re-suspended in DMEM for transplantation.

### Rat spinal cord transection and SC bridge implantation

While anesthetized, the rats received a laminectomy from thoracic vertebrae levels 7 (T7) to T9, with significant lateral exposure, and the dorsal roots were cut. Then a single incision was made with angled micro-scissors to completely transect the spinal cord at T8. The ventral dura and spinal roots were severed whereupon the spinal cord stumps retracted to create a 2–3 mm gap. Completeness of the transection was confirmed by lifting the rostral and caudal stumps and placing them into the ends of a 5.0 mm long polyacrylonitrile/polyvinyl chloride (PAN/PVC) channel (kindly provided by Dr. Tresco, University of Utah). Then, 3.0 X 10^6^ SCs in 15 μL DMEM were mixed with 10 μL of Matrigel (BD Biosciences, San Jose, CA) and injected through the rostral of two holes previously created in the top of the channel.

### Rat tissue processing, immunohistochemistry, and imaging

The rats received intraperitoneal injections of terminal anesthesia (ketamine, 200 mg/kg and xylazine, 20 mg/kg), their left ventricles were injected with 20 USP units of heparin (Sigma, St. Louis, MO), and they were subjected to transcardial perfusion with 200 mL 4°C, phosphate-buffered saline (PBS, Invitrogen) followed by 400 mL 4°C, 4% paraformaldehyde (PFA, Sigma) in 0.1 M phosphate buffer (pH 7.4). Spinal cords were extirpated and further fixed in PFA overnight and cryoprotected in PBS plus 30% sucrose and 0.025% sodium azide (Sigma). Tissue blocks were embedded in PBS plus 12% gelatin (Sigma) and 0.025% sodium azide and quick frozen in crushed dry ice. The SC bridges with attached rostral and caudal spinal cord were sectioned sagittally from left to right at 20 μm using a cryostat (Leica, Buffalo Grove, IL) and mounted directly onto a series of five slides (Surgipath, Buffalo Grove, IL). In this way sections were mounted onto a slide series at 100 μm intervals.

The antigenic sites were blocked in PBS with 0.5% Triton-X (Sigma) plus 5.0% normal goat serum (Atlanta Biological, Lawrenceville, GA) and/or 5.0% normal donkey serum (Atlanta Biological). Then, tissue sections were incubated overnight with one or more of the following primary antibodies: GFP (chicken, 3.19 mg/mL, 1:500, Chemicon, Temecula, CA), dopamine beta-hydroxylase (DβH, mouse, 1:500, Chemicon), glial fibrillary acidic protein (GFAP, SMI 22, mouse, 1:500, Covance, Denver, PA; rabbit, 1:500, DAKO, Carpinteria, CA), S100 (rabbit, 1:500, DAKO), and MASH1 (rabbit, 1:200, Santa Cruz, CA), together with secondary antibodies conjugated to 488 or 594 fluorophores (1:200, Molecular Probes, Eugene, OR) or Cy-5 (1:200, Jackson ImmunoResearch, West Grove, PA), as well as 0.1% Hoechst dye solution (10 mM) to label nuclei. All images of immunostaining were obtained with an Olympus FV1000 confocal microscope.

### Quantification of spinal cord axon regeneration

Adhering to guidelines established for the assessment of axon regeneration [33], sagittal sections of the SC bridge were analyzed by a line-transect method using Neurolucida (MBF Bioscience, Williston, VT) and an Axiophot fluorescent microscope (Zeiss, Thornwood, NY) and MAC 5000 XYZ stage (Ludl, Hawthorne, NY). The extent of host spinal cord tissue inside the polymer channel was defined by the expression of GFAP-positive astrocyte somata at the spinal cord/SC bridge interfaces. The zero point, 0’, was determined on the tissue section that contained the tip of rostral host spinal cord inserted farthest into the polymer channel. This distance from the rostral end of the polymer channel was then used to locate 0’ on the remaining sections. Then, using Hoechst-staining to visualize the tissue, dorso-ventral lines were drawn on each section along the rostro-caudal axis at −10.0 mm, 0.25 mm, 0.5 mm, 1.0 mm, 1.5 mm, 2.0 mm, and 2.5 mm from 0’. The length of these lines at a given location indicated the dorso-ventral thickness of bridge tissue. By focusing up and down through the entire tissue section, the numbers of DβH-positive axons or GFAP-positive processes that crossed a given dorso-ventral line were quantified. In this way, a transverse plane of tissue was analyzed and the area was determined by multiplying the tissue section thickness (20 μm) by the length of the dorso-ventral lines at a given location. To normalize for differences in the amount of bridge tissue sampled across animals, this area was used to report counts as DβH-positive axons/mm^2^ or GFAP-positive processes/mm^2^.

### Assessment of rat hindlimb locomotor function

To assess hindlimb motor function, animals were subjected to a four minute open-field BBB locomotor test [34]. All rats were tested one week before the stereotaxic injection surgery and one week before the transection/bridge implantation. Their bladders were expressed prior to testing. The rats were then tested on a weekly basis, for six weeks after the transection/bridge implantation. In the final week, the rats also were recorded with a video camera while being tested. To enhance the metric properties of the scale, the scores were converted from the 21 point scale to a modified 12 point scale [35]. The modified 12 point scale pools scores on the 21 point scale. The scores that are pooled are ones that are rarely assigned to an injured animal, and therefore create a problem for both parametric and non-parametric analysis. For example, scores above 14 (rarely assigned) on the 21 point scale are pooled together. Pooling scores in this manner is most appropriate when using the BBB behavioral test to assess function after severe spinal cord injury.

### Statistics

All statistical analyses were performed with the Graph Pad Prism 5 software. Mann-Whitney T-tests and a one-way ANOVA with a Bonferroni post hoc test were used to analyze zebrafish qPCR and axon regeneration data, respectively. A two-way ANOVA with a Bonferroni post hoc test was used to compare axon quantification data and BBB test scores from control and treated rats (+/− indicates SEM). A Fisher’s exact test was used to analyze rat BBB test scores at week six.

## Results

### Mash1 is required for gap43 expression after optic nerve injury in zebrafish

A 3.6 kb genomic sequence directly upstream of the pufferfish (fugu), *gap43* coding sequence is sufficient to induce EGFP reporter expression during optic nerve regeneration in zebrafish [26]. Within the 3.6 kb fugu sequence there are specific regions necessary to activate gene expression during axon regeneration [18]. These regions are highly conserved among teleosts and include putative E-box binding motifs for class II bHLH proteins such as Ascl1a. Therefore, MOs previously characterized to knockdown Ascl1a in the zebrafish retina [22] were utilized to determine if Ascl1a regulates the expression of the fugu *gap43* driven *egfp* transgene after optic nerve injury.

**Fig 1:**
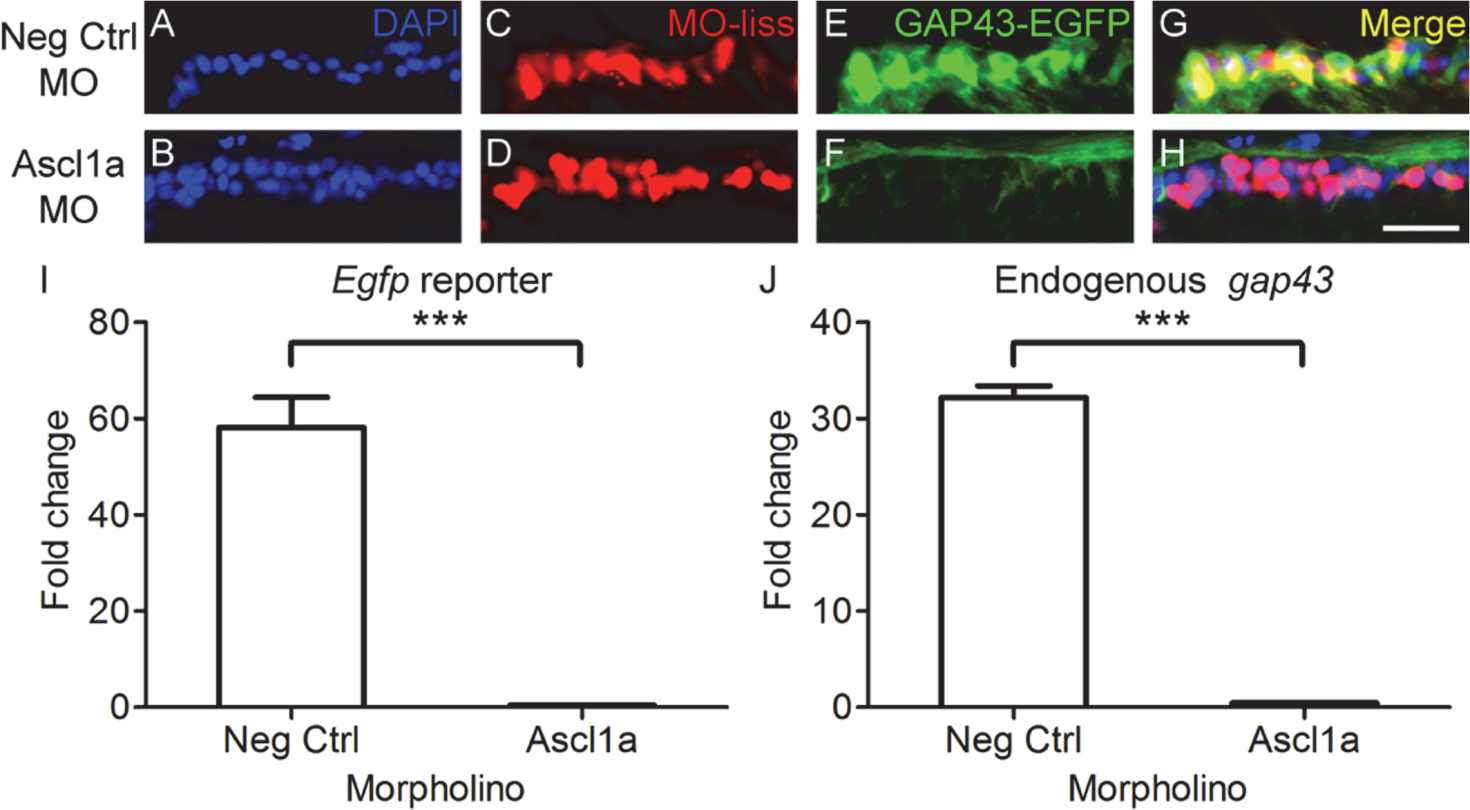
Ascl1a is required for *gap43 gene* expression in zebrafish. A-H, Cross sections through the retina focused on the retinal ganglion cell layer. A-D, Strong induction of *gap43* expression, visualized by the EGFP reporter (C, green; D, yellow), is observed after optic nerve transection in RGCs receiving negative control MOs tagged with lissamine (MO-liss; B, red). In contrast, injury-induced *gap43* expression is greatly reduced (G, green; H, yellow) in RGCs receiving Ascl1a MOs tagged with lissamine (F, red). DAPI stained nuclei are blue (scale bar = 5 μm). I, J, Relative fold change over unlesioned control represented graphically. Quantitative real-time PCR demonstrated that there was a reduction in both *egfp* reporter gene expression (I) and endogenous *gap43* expression (J) in *gap43*:*egfp* zebrafish that received Ascl1a MOs (n = 4) after optic nerve transection compared to those that received negative control MOs (n = 4; *** = p<0.001, Mann-Whitney T-test).

After adult *gap43*:*egfp* zebrafish received a transection of the optic nerve, MOs were acutely applied to the site of injury. Red fluorescence of the lissamine tag confirmed retrograde transport of the MOs to RGC somata. Transgene expression was then assessed four days after injury and application of the MOs. Normally, upon optic nerve injury in *gap43*:*egfp* zebrafish, RGCs display a strong induction of *gap43*:*egfp* transgene expression [18]. In contrast, injured RGCs with Ascl1a knockdown showed a dramatic reduction in the expression of the *gap43*:*egfp* transgene (Fig. 1A-H). To quantify the relative differences in transgene expression, qPCR was performed. This demonstrated that when *gap43*:*egfp* zebrafish received Ascl1a MOs after optic nerve injury, there was a greater than 50-fold reduction in EGFP reporter expression compared to negative controls (p<0.001; Fig. 1I). The absence of caspase co-localization to RGCs and presence of protein HuC/HuD (S2 Fig.) suggests that the reduction in transgene expression post Ascl1a knockdown was due to regulation by Ascl1a and not RGC cell death. Therefore, Ascl1a is required for induction of gap*43* gene expression after optic nerve injury in zebrafish.

Given the high degree of conservation in GAP43 promoter/enhancer regions across distantly related teleost species [26], injury induced expression of the endogenous zebrafish *gap43* gene was predicted to demonstrate the same dependence on Ascl1a activity as was shown above for the fugu *gap43* driven transgene. Compared to controls, qPCR analysis confirmed that the relative levels of endogenous zebrafish *gap43* expression was almost completely abolished in animals that received Ascl1a MOs (p<0.001; Fig. 1J). Together these results demonstrate that Ascl1a regulation of *gap43* gene in RGCs after optic nerve injury is conserved in teleost species that diverged approximately 300 million years ago [36].

### Ascl1a is required for retinal ganglion cell axon regeneration in zebrafish

**Fig 2:**
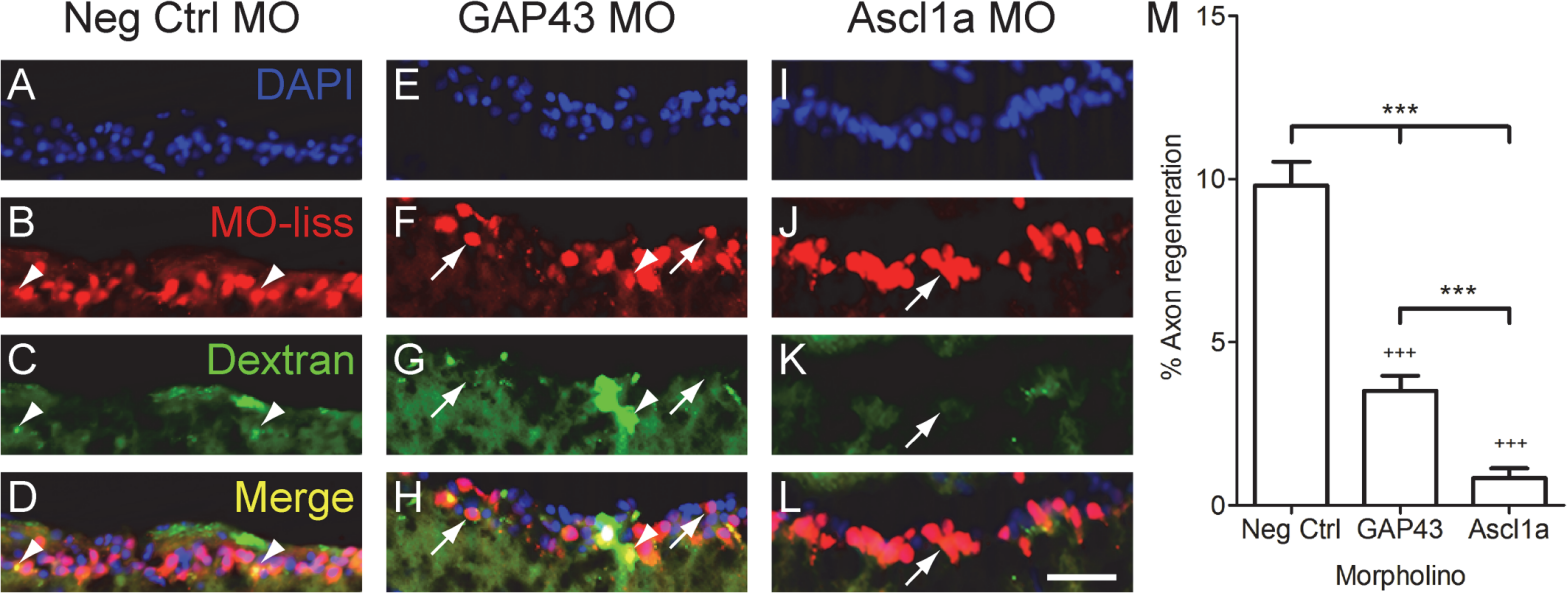
Ascl1a is required for RGC axon regeneration in zebrafish. A-L, Cross sections through the retina focused on the retinal ganglion cell layer. Four days after optic nerve transection, zebrafish RGCs that received MOs (red, arrowheads) were retrogradely traced with dextran (green) 1 mm beyond the site of injury. Animals that received negative control MOs (A-D) were able to regenerate RGC axons as evident by the co-localization of MOs and tracer (yellow, arrow). In contrast few RGCs regenerated axons in animals that received MOs to knockdown GAP43 (E-H) or Ascl1a (I-L). DAPI stained nuclei are blue (scale bar = 10 μm). M, Quantification of the percentage of RGCs that received the MOs and were able to regenerate axons. Compared to controls (n = 6), those that received either GAP43 (n = 6) or Ascl1a (n = 6) MOs exhibited a reduced percentage of RGC axons to regenerate (*** = p<0.001 one-way ANOVA; +++ = p<0.001 Bonferroni posttest). Compared to animals that received GAP43 MOs, those that received Ascl1a MOs exhibited a greater reduction in the percentage of RGC axons to regenerate (*** = p<0.001, t-test).

To determine if Ascl1a is required for axon regeneration in teleosts, we used morpholinos to acutely knock down Ascl1a expression in RGCs of adult zebrafish after optic nerve crush. Percent regeneration was compared between fish treated with MOs targeting Ascl1a, GAP43, or a negative control MO. Four days after injury, RGCs that regenerated axons 1 mm beyond the site of injury were retrogradely labeled and counted. In control animals, approximately 10% of the injured axons grew beyond the injury site by four days after injury (Fig. 2A-D). However, animals that received MOs to knockdown GAP43 (Fig. 2E-H) or Ascl1a expression (Fig. 2I-L) exhibited a reduced percentage of regenerating axons compared to controls (p<0.001; Fig. 2M). Furthermore, compared to animals that received MOs for GAP43, those that received MOs for Ascl1a exhibited a greater reduction in the percentage of regenerating axons, with less than 1% growing 1 mm past the injury site (p<0.001; Fig. 2M). Together these data demonstrate that Ascl1a is required for axon regeneration after optic nerve injury in zebrafish and suggests that this is accomplished through the regulation of multiple regeneration-associated genes.

### AAV vectors induce the expression of MASH1 in adult rat brainstem neurons

**Fig 3:**
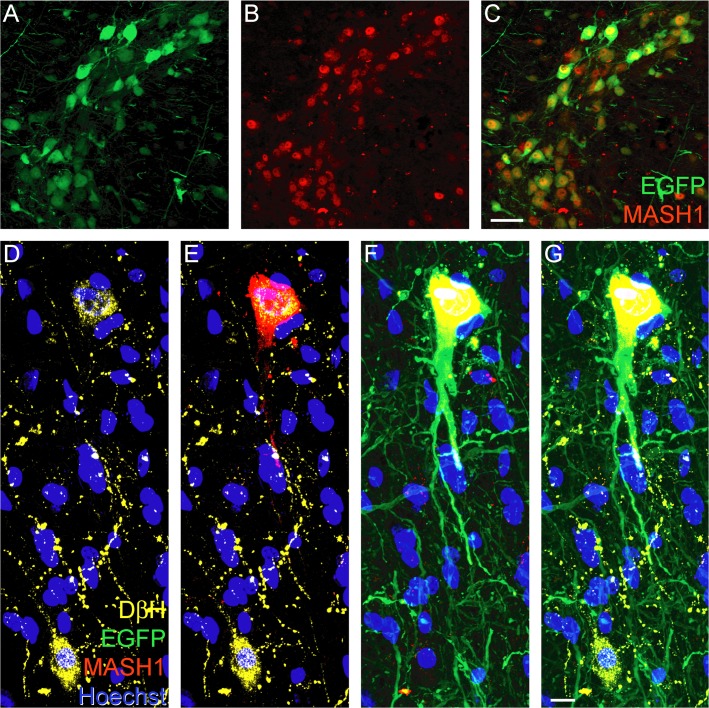
Injection of AAV-EGFP plus AAV-MASH1 induces expression of MASH1 in adult rat brainstem neurons. Immunostaining for EGFP (A, green) and MASH1 (B, red) revealed co-localization in brainstem neurons (C, yellow, scale bar = 20 μm). D, Immunostaining for DβH (yellow) revealed two noradrenergic neurons with Hoechst staining (blue) to visualize nuclei. E, Immunostaining for MASH1 (red) showed that the protein is expressed in the upper DβH-positive neuron. F, MASH1 was only expressed in neurons that also expressed EGFP (green). G, A merged image showed that the lower DβH-positive neuron was not infected with AAV and consequently did not express EGFP or MASH1 (scale bar = 5 μm).

AAV vectors were developed to determine if MASH1 expression can induce axon regeneration in young adult rats. Given the fundamental role for MASH1 in the development of noradrenergic neurons [20], stereotaxic injections of AAV were performed, as described by Williams and colleagues [30], to target populations of those neurons in the locus coeruleus, ventro-lateral pons, and ventro-medial medulla in the rat. In treated animals, injected with AAV-EGFP plus AAV-MASH1, this resulted in the co-expression of MASH1 and EGFP in numerous brainstem neurons (Fig. 3A-C), including noradrenergic neurons that expressed DβH (Fig. 3D-G). Neither EGFP nor MASH1 was detected in non-neuronal cells such as astrocytes, as determined by an absence of co-expression with GFAP. MASH1 protein was typically located in the nucleus of neurons, although some exhibited cytosolic localization and/or low levels of expression. MASH1 protein expression was observed in treated animals for the entire duration of the study and was not expressed in animals injected with AAV-EGFP only (controls).

### MASH1 promotes noradrenergic brainstem axon regeneration in rats

The complete transection of the thoracic spinal cord and implantation of a SC bridge offers a rigorous paradigm to assess axon regeneration. Historically, supraspinal axons have not been found to regenerate following complete transection of the thoracic spinal cord and implantation of either a peripheral nerve graft or SCs [2,37]. However, a recent modification of the complete transection/SC bridge paradigm demonstrated that when GFAP+ processes extend into the implant, brainstem axons are able to regenerate [38]. Therefore, the current study utilized the same technique to determine if the expression of MASH1 in noradrenergic brainstem neurons will enhance the ability of their axons to regenerate after injury.

**Fig 4:**
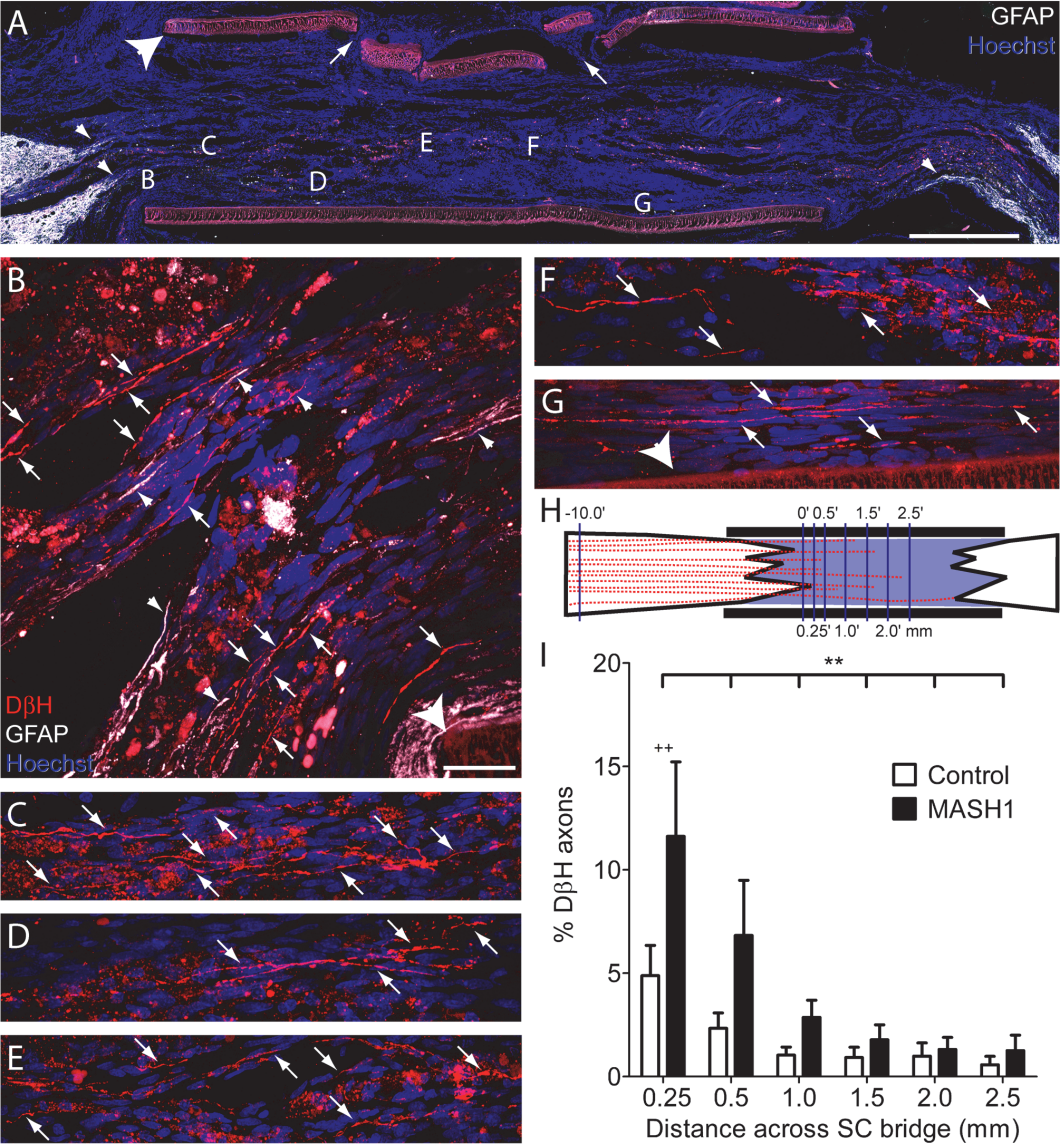
Rats treated with MASH1 exhibit increased regeneration of noradrenergic axons into the SC bridge. A, A low magnification image of a SC bridge from a MASH1 treated animal showing the rostral (left) and caudal (right) spinal cord/SC bridge interfaces delineated by GFAP-positive astrocytes (white). Note the long astrocyte processes (small arrowheads, A and B) that extend into the bridge. Hoechst-stained nuclei are blue and the large arrowhead indicates the polymer channel. Small holes (arrows) in the top of the channel were created to inject a fluid mixture of SCs and Matrigel (scale bar = 1 mm). Locations of higher magnification images taken on an adjacent section are indicated by letters (B-G). In treated animals, many beaded DβH-positive axons (red, arrows) regenerated 0.25 mm (B), 0.5 mm (C), 1.0 mm (D), 1.5 mm (E), 2.0 mm (F), and more than 2.5 mm (G), beyond the rostral spinal cord/SC bridge interface (scale bar = 20 μm). H, An illustration of the line-transect method of analysis, depicting DβH-positive axons (red) regenerating into a SC bridge. The polymer channel (thick black lines) and the transverse dorso-ventral lines used for quantification (thin purple lines) are diagrammed. The numbers represent mm from the rostral interface. I, The percentage of DβH-positive axons 10 mm rostral to the bridge that regenerated across the bridge was greater in MASH1 treated animals (n = 10) compared to control animals (n = 11; ** = p<0.01, two-way ANOVA; ++ = p<0.01 Bonferroni posttest).

Following the complete transection of the thoracic spinal cord and implantation of a SC bridge, we observed numerous DβH-positive brainstem axons to regenerate in animals treated with MASH1 (Fig. 4A-G). The line-transect method of analysis (Fig. 4H) was utilized to quantify this regeneration at progressive distances from the rostral spinal cord/SC bridge interface in both control and MASH1 treated animals. This revealed that a greater number of DβH-positive axons regenerated into the SC bridge in MASH1 treated animals (p<0.001, two-way ANOVA). At 0.25 mm past the rostral spinal cord/SC bridge interface, an average of 98 +/− 26 DβH-positive axons/mm^2^ were found to regenerate in MASH1 treated animals compared to only 38 +/− 11 DβH-positive axons/mm^2^ in control animals (p<0.01, Bonferroni Posttest). The numbers of DβH-positive axons also were quantified 10 mm rostral to the bridge, revealing no difference between control and MASH1 treated animals (796 +/− 77 axons/mm^2^ versus 869 +/− 40 axons/mm^2^, respectively). The axons quantified 10 mm rostral to the bridge were then used to determine the percentage of axons that regenerated in a given animal. This also demonstrated that a greater percentage of DβH-positive axons regenerated in animals treated with MASH1 compared to controls (Fig. 4I; p<0.005; two-way ANOVA). As many as 38% of the DβH-positive axons counted rostral to the bridge regenerated following MASH1 treatment, compared to at most 13% in control bridges. Although DβH-positive axons crossed the entire bridge (2.5 mm), they were not observed to regenerate into the caudal spinal cord, distal to the bridge.

**Fig 5:**
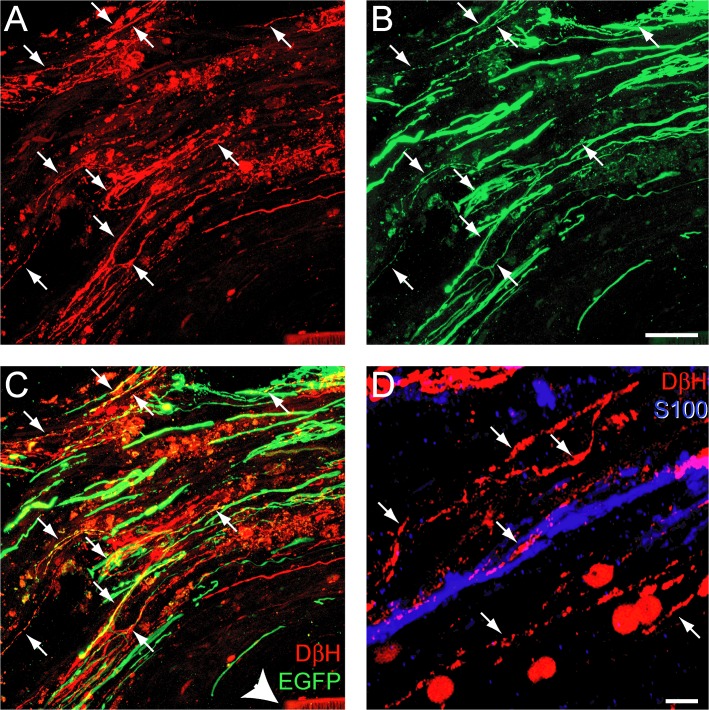
Rats treated with MASH1 exhibit EGFP expression in noradrenergic axons that regenerated into a SC bridge. A-C, Expression of DβH (red) and EGFP (green) was sometimes co-localized (arrows) in axons that regenerated past the rostral spinal cord/SC bridge interface. Arrowhead indicates the polymer channel (scale bar = 20 μm). D, DβH-positive axons (red, arrows) regenerated in close proximity to S100-positive SCs (blue; scale bar = 5 μm).

In MASH1 treated animals, DβH-positive axons that regenerated into the bridge expressed EGFP (Fig. 5A-C) indicating the expression of MASH1 in their neuronal somata. However, as described previously by Williams and colleagues [30], a direct comparison of EGFP-labeled brainstem axons between groups was not possible because control animals expressed 2x more EGFP than those treated with MASH1. At the rostral interface, DβH-positive axon regeneration was often in close association with GFAP-positive processes entering the bridge (Fig. 4A and B) and SCs (Fig. 5D). In order to normalize for potential differences in the rostral host/SC bridge interface, the line-transect method was utilized to quantify the number of GFAP-positive processes in the transplant [38]. This demonstrated that there was no difference between the total number of GFAP-positive processes at 0.5 mm and 1.0 mm distal to the rostral interface in control and treated animals (42 +/− 26 processes/mm^2^ versus 50 +/− 14 processes/mm^2^, respectively). Therefore, the observed differences in axon regeneration between groups are not due to differences in the rostral host spinal cord/SC bridge interface. In summary, the expression of MASH1 directly increased both the total number and the percent of noradrenergic axons that regenerated into a SC bridge

### MASH1 expression in brainstem neurons improves hindlimb joint movements in rats

**Fig 6:**
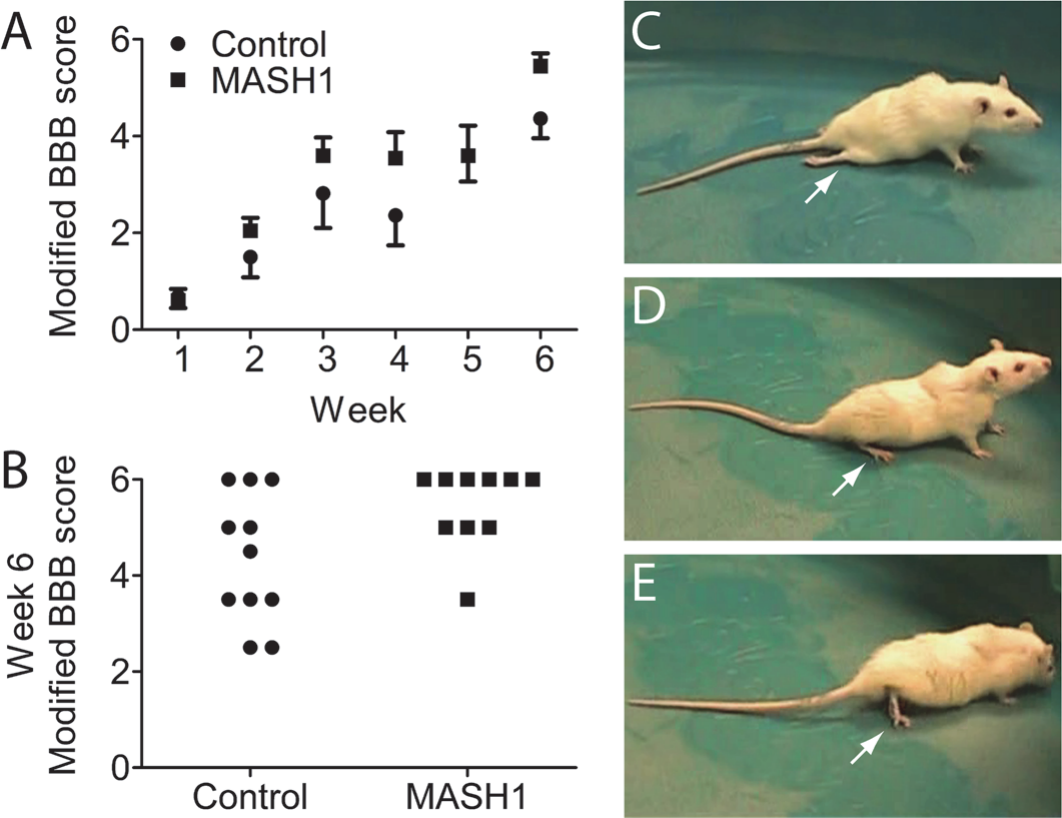
Rats treated with MASH1 exhibit improved hindlimb joint movements. A, MASH1 treated animals (n = 10) exhibited improved scores over all six weeks on the modified BBB scale compared to controls (n = 11; p<0.05; two-way ANOVA; error bars = SEM). The average scores for control and treated animals at week five were the same, although the SEMs were different. B, At week six, 90% of MASH1 treated animals demonstrated extensive movement of all three hindlimb joints in both legs compared to only 45% of the controls (p<0.05; Fisher’s exact test). Still-frame images from the week 6 BBB test for a control animal (C) and a MASH1 treated animal (D, E): control animal hindlimbs were often flaccid and without movement as depicted in C (arrow) whereas, in contrast, MASH1 treated animals displayed plantar placement and stepping as depicted in D and E (arrows).

Following the complete transection and implantation of a SC bridge, rat hindlimb joint movements were analyzed on a weekly basis using the BBB test [34]. Throughout the six weeks after injury, a BBB scale modified to assess severe spinal cord injuries [35] revealed improved hindlimb movements in MASH1 treated animals compared to controls (Fig. 6A; p<0.05). At week six, 90% of the treated animals exhibited extensive movement of all three joints in both hindlimbs (BBB score ≥ 5) compared to only 45% of the controls (Fig. 6B; p<0.05). The differences in hindlimb movements between the two groups were easily discernible in videos taken during BBB testing. The majority of control animals exhibited minimal movement and/or flaccid hindlimbs (Fig. 6C). In contrast, MASH1 treated animals often exhibited crawling and/or plantar placement (Fig. 6D, E).

## Discussion

By utilizing different models of CNS injury across vertebrates, the present study demonstrates that MASH1 expression is an evolutionarily conserved mechanism necessary for axon regeneration in a teleost that can be ectopically induced to enhance axon regeneration in a mammal. In contrast to teleosts, following injury to the mammalian CNS, both extrinsic and intrinsic treatments will be required to induce axon regeneration and substantial functional recovery [2,39]. Results presented here not only support the notion that combination strategies are needed to repair the injured mammalian CNS, but demonstrate a novel proof-of-principle to alter the intrinsic state of brainstem neurons. The expression of a transcription factor, normally only transiently present during mammalian development, may revert adult CNS neurons to a state where they are capable of axon regeneration and functional repair.

Experiments with transgenic zebrafish elucidated mechanisms whereby MASH1 alters the intrinsic state of adult neurons and promotes axon regeneration. Through analysis of the GAP43 promoter, several potential sites of regulation were identified previously in zebrafish, including consensus E-box binding motifs [16–18], to which Class II bHLH proteins, such as Ascl1a, may bind. In the current study, knockdown of Ascl1a expression in zebrafish RGCs after optic nerve transection reduced both *gap43* gene expression and axon regeneration. Therefore, this likely occurred through Ascl1a directly binding to the E-box motifs. Furthermore, given that the knockdown of Ascl1a reduced RGC axon regeneration greater than the knockdown of GAP43 alone, Ascl1a probably promotes axon regeneration through the regulation of other genes in addition to GAP43, such as alpha Tubulin [22]. This is supported by previous reports that MASH1 regulates axon regeneration associated genes such as c-Ret [40,41], Ndrg4 [41], HuD [42], and NogoR [43]. The use of chromatin immunoprecipitation assays also demonstrates that MASH1 directly binds to the cis-regulatory regions of genes associated with axonogenesis and axon guidance [21], including the RhoA inhibitor, Rnd3 [44]. Therefore, Ascl1a/MASH1 appears to be a fundamental component of transcriptional complexes that promote axon regeneration.

MASH1 is expressed during the development of several neuronal populations, including noradrenergic neurons [20]. Unlike in adult zebrafish, MASH1 is not expressed after axonal injury in the adult rat CNS. To determine if MASH1 could promote axon regeneration in a mammal, AAV vectors were used to ectopically express MASH1 in noradrenergic brainstem neurons of adult rats. After complete transection of the thoracic spinal cord and implantation of a SC bridge, MASH1 led to increased noradrenergic axon regeneration into the SC bridge. Thus, MASH1 was able to induce axon regeneration in the same population of rat CNS neurons that transiently express MASH1 during development. The genes regulated by MASH1 in mammals are likely similar to those in teleosts given the conserved E-box motifs present in the promoter regions of genes associated with axon regeneration [18,45]. Thus, MASH1 may be converting adult CNS neurons to a developmental state that is capable of axon regeneration, not unlike the ability of MASH1 and other Class II bHLH proteins to reprogram various somatic cell types [46–57].

Although MASH1 exerts a robust effect on axon regeneration in the zebrafish, the observed effects in the mammalian CNS were modest. In addition to differences in the injury environment that may deter axon regeneration to a greater extent in mammals compared to lower vertebrates, intrinsic factors may be limiting the effects of MASH1 expression. One possibility is that Mash1 has reduced DNA accessibility due to a closed chromatin state. Hence co-expression with molecules/pathways that relax the chromatin such as p300 [58] may be required to form transcriptional complexes that are more capable of inducing changes in gene expression. In addition, the efficacy of such complexes also may be improved through manipulations of miRNA [56,59–61]. Noteworthy is that MASH1 protein was not robustly expressed in all brainstem neurons infected with AAV-EGFP plus AAV-MASH1. Given that EGFP expression was not uniform across neurons, it could be that viral transduction was not equally successful in all neurons. In addition, negative regulators may limit MASH1 protein expression in adult CNS neurons. For example, Class V and VI bHLH proteins, Id and HES family members, respectively, are up-regulated after SCI [62] and may sequester ubiquitous class I binding partners from other bHLH proteins [63–67]. Of these, Id1 [68–70] and Id2 [65] limit the activity of MASH1, and Id2 decreases expression of GAP43 [45] and TrkB [71]. Id2, however, may be required for MASH1 expression [72], to induce axonogenesis [73], and to improve the growth of injured sensory axons [74]. Given that Id proteins inactivate neurogenic repressors such as HES-1 [65], MASH1 and Id2 may function to re-program adult neurons by out-competing or sequestering, respectively, bHLH proteins that limit axon regeneration.

The observed improvement in hindlimb movements in MASH1 treated animals is consistent with the largest improvements observed in previous studies of complete thoracic spinal cord transection. There currently appears to be a “ceiling” for behavioral recovery following thoracic spinal cord injury that is likely due to the absence of axons being directly observed to regenerate across the distal interface and into the caudal spinal cord [75]. The improved behavioral scores in MASH1 treated animals are likely due to the increased regeneration of noradrenergic and/or potentially other brainstem neurons to the distal end of the bridge. These axons may have formed synapses with dendrites present at the caudal SC bridge/spinal cord interface as previously implicated by Williams and colleagues [38]. In this way, synaptic and/or extrasynaptic release of neurotransmitters at the distal interface could potentially influence central pattern generator activity [76] and thereby improve hindlimb movements to a greater extent in animals treated with MASH1.

In conclusion, Ascl1a/Mash1 expression is required for axon regeneration in zebrafish and enhances axon regeneration and functional recovery in rats. While both mammals and teleosts undergo a developmental switch in which growth-associated genes are transcriptionally silenced, in zebrafish these genes are reactivated upon CNS injury, in part dependent upon Ascl1a. Thus, the mechanisms by which bHLH proteins regulate growth-associated genes after injury were not conserved across vertebrates and this may explain the limited ability for mammalian CNS neurons to regenerate their axons. Understanding the evolutionarily conserved and developmental mechanisms that mediate a neuron’s intrinsic ability to recapitulate axonal growth will reveal new treatment strategies for CNS repair and recovery of function.

## Supporting Information

S1 Dataset Data for optic nerve transection and spinal cord injury experiments. The first dataset (Fig. 1I) represents the relative fold change in EGFP gene expression over unlesioned controls for each experimental negative control or Ascla knockdown animal. The second dataset (Fig. 1J) represents fold change in GAP43 gene expression over unlesioned controls for each experimental negative control or Ascla knockdown animal. The third dataset (Fig. 2M) represents the percentage of RGCs that received the MOs and were able to regenerate axons for each experimental control or Ascla knockdown animal. The fourth dataset (Fig. 4I) represents percentage of DβH-positive axons that were able to regenerate to a given distance for each experimental control or MASH1 treated animal. The last dataset (Fig. 6) represents weekly hindlimb joint movement scores (modified BBB scores), for each experimental control or MASH1 treated animal.
(XLSX)

S1 Fig GAP43 MOs reduce the expression of GAP43-EGFP after optic nerve transection. The expression of the GAP43-EGFP fusion protein (green) in *fgap43*:*egfp* zebrafish RGCs after optic nerve transection and the application of gel foam soaked with GAP43 MOs tagged with lissamine (red, A-D) or in a control animal that received gel foam only, and no MOs (E). DAPI stained nuclei are blue in A and D (scale bar = 5 μm).
(TIF)

S2 Fig Reduction in transgene expression in RGCs post Ascl1a knockdown is due to regulation by Ascl1a and not cell death. The expression of caspase (purple, A-C) and HuD (purple, D-E) in zebrafish RGCs after optic nerve transection and application of gel foam soaked with Ascl1a MOs tagged with lissamine (red). DAPI stained nuclei are blue in A and D (scale bar = 5 μm).
(TIF)
