# Supplementary figures and images for: MASH1/Ascl1a Leads to GAP43 Expression and Axon Regeneration in the Adult CNS
Source: PLoS One. 2015 Mar 9;10(3):e0118918. doi: 10.1371/journal.pone.0118918 (PMC4353704; doi:10.1371/journal.pone.0118918)

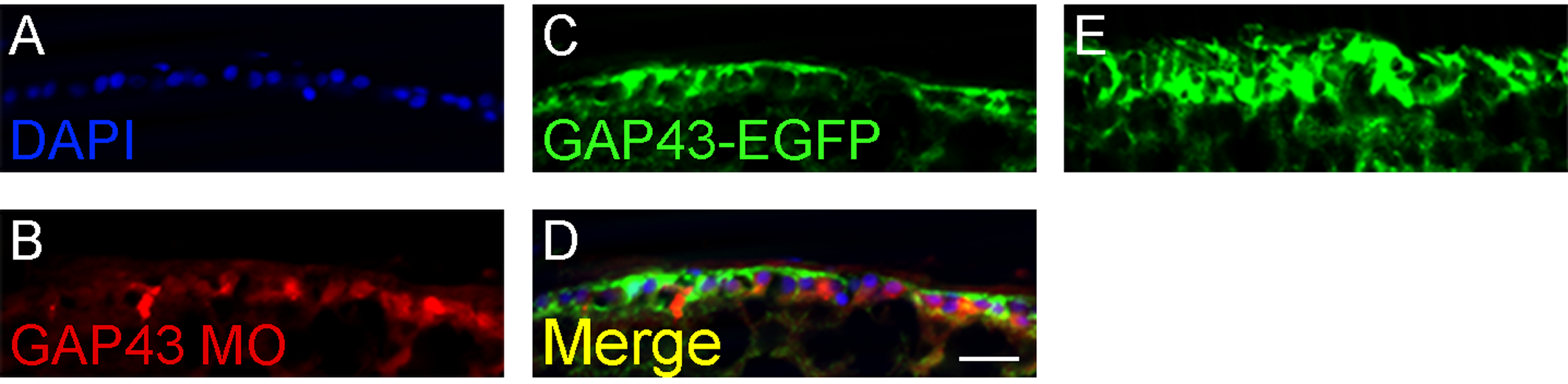

Supplement: S1 Fig — The expression of the GAP43-EGFP fusion protein (green) in fgap43:egfp zebrafish RGCs after optic nerve transection and the application of gel foam soaked with GAP43 MOs tagged with lissamine (red, A-D) or in a control animal that received gel foam only, and no MOs (E). DAPI stained nuclei are blue in A and D (scale bar = 5 μm). (TIF) [file pone.0118918.s002.tif]

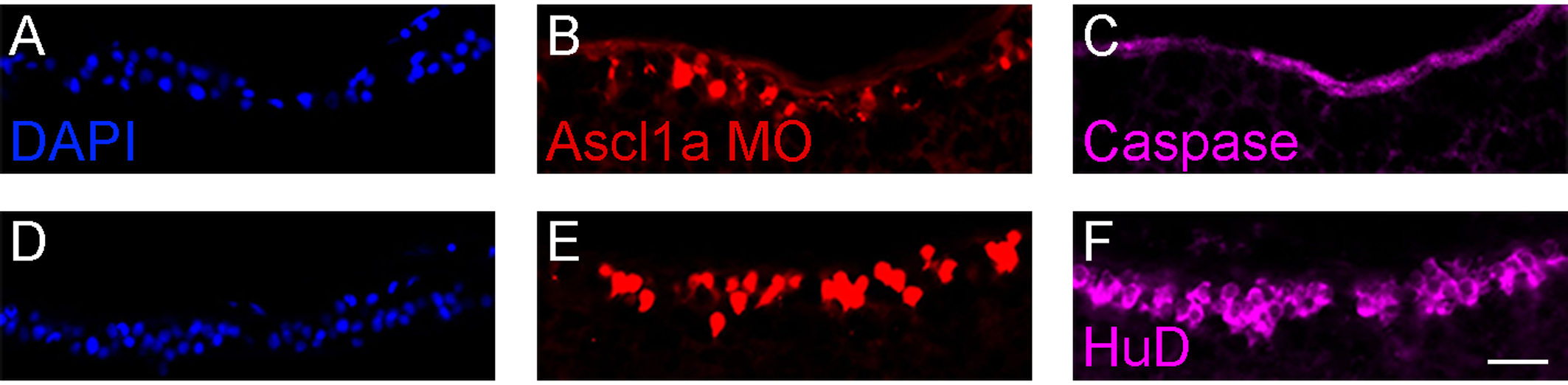

Supplement: S2 Fig — The expression of caspase (purple, A-C) and HuD (purple, D-E) in zebrafish RGCs after optic nerve transection and application of gel foam soaked with Ascl1a MOs tagged with lissamine (red). DAPI stained nuclei are blue in A and D (scale bar = 5 μm). (TIF) [file pone.0118918.s003.tif]
